# Health-related quality of life after vertebral or hip fracture: a seven-year follow-up study

**DOI:** 10.1186/1471-2474-10-135

**Published:** 2009-11-03

**Authors:** Inger Hallberg, Margareta Bachrach-Lindström, Staffan Hammerby, Göran Toss, Anna-Christina Ek

**Affiliations:** 1 Department of Medical and Health Sciences, Division of Nursing Science, Faculty of Health Sciences, Linköping University, SE-581 85 Linköping, Sweden; 2 Department of Medical and Health Sciences, Division of Radiological Science, Faculty of Health Sciences, Linköping University, SE-581 85 Linköping, Sweden; 3 Department of Medical and Health Sciences, Division of Cardiovascular Medicine/Internal Medicine, Faculty of Health Sciences, Linköping University, SE-581 85 Linköping, Sweden; 4 Department of Endocrinology & Gastroenterology University Hospital/Osteoporosis Unit, SE-581 85 Linköping, Sweden

## Abstract

**Background:**

The negative impact of vertebral and hip low-energy fractures on health-related quality-of-life (HRQOL) has been demonstrated previously, but few prospective long-term follow-up studies have been conducted. This study aims to (i) investigate the changes and long-term impact of vertebral or hip fracture and between fracture groups on HRQOL in postmenopausal women prospectively between two and seven years after the inclusion fracture, (ii) compare HRQOL results between fracture and reference groups and (iii) study the relationship between HRQOL and physical performance, spinal deformity index and bone mineral density at seven-year follow-up.

**Methods:**

Ninety-one women examined two years after a low-energy vertebral or hip fracture were invited to a new examination seven years after the diagnosis. HRQOL was examined using the SF-36 questionnaire and was compared with an age and sex-matched reference group. Physical function was assessed using tests and questionnaires. Bone mineral density was measured. Radiographs of the spine were evaluated using the visual semiquantitative technique. A longitudinal and cross-sectional design was used in this study. Statistical analyses included descriptive statistics, Student's *t*-tests, ANCOVA, and partial correlation.

**Results:**

Sixty-seven women participated. In the 42 women (mean age 75.8, SD 4.7) with vertebral fracture as inclusion fracture, bodily pain had deteriorated between two and seven years and might be explained by new fracture. Remaining pronounced reduction of HRQOL was seen in all domains except general health and mental health at seven-year follow-up in women with vertebral fractures compared to the reference group (p < 0.05). All 25 women (mean age 75.0, SD 4.7) with hip fracture as inclusion fracture had no significant changes in HRQOL between two and seven years and did not differ from the reference group regarding HRQOL after seven years. The vertebral group had significantly lower values for bodily pain, vitality, role-emotional function and mental health compared to the hip group. HRQOL showed a positive relationship between physical activity, static balance and handgrip strength.

**Conclusion:**

The long-term reduction of HRQOL in women with vertebral fracture emerged clearly in this study. The relationships between HRQOL and physical performance in women with vertebral and hip fracture raise questions for more research.

## Background

The global burden of osteoporosis includes considerable numbers of fractures, morbidity, mortality and expenses, due mainly to vertebral, hip and forearm fractures [1-4]. Osteoporosis causes no symptoms except for fractures and their complications. All fractures may lead to disability or impairment of health-related quality of life (HRQOL), particularly those of the hip and vertebrae [5-7]. Vertebral fractures are the most common of all osteoporotic fractures, and underdiagnosis of vertebral fracture is a worldwide problem [8]. Furthermore, vertebral and hip fractures are linked to increased mortality [9-12].

Several studies have shown more or less severe impairment of HRQOL in patients who have experienced vertebral or hip fractures [5]. The extent to which the impairment of HRQOL is due to fractures or other co-morbidity or biological ageing is not known. In elderly people with osteoporosis, impairment of balance has been reported [13]. A recent study has also reported that balance impairment is related more to vertebral fracture than to thoracic kyphosis in women with osteoporosis [14]. Handgrip strength is necessary for performing activities of daily living and is essential for maintaining functional autonomy, and may also mirror ageing and frailty. Fractures and pain are independently related to lower handgrip strength and walking speed [15]. Further studies are needed on the role of these factors in HRQOL after fractures.

Vertebral fracture can be classified into two major categories, subclinical and clinical. Studies of patients with subclinical as well as clinical vertebral fractures show association with decrements in function and HRQOL. The decrement is greater when the number of fractures is higher and the severity is greater [16,17].

It is noteworthy that only a few clinical trials have shown treatment benefits regarding HRQOL [18-21]. Several recent cross-sectional studies [22-25] and some follow-up studies [26-30] of HRQOL after fracture have been published, but the long-term impact of osteoporotic fractures on HRQOL has not been prospectively or sufficiently examined. In a previous study [29] we reported that vertebral and hip fractures have a considerably greater and more prolonged impact on HRQOL than do forearm and humerus fractures. HRQOL was significantly reduced at baseline regarding all SF-36 domains after vertebral and mostly hip fracture, but only regarding some domains after forearm and humerus fracture. However, two years after hip fracture, HRQOL had improved but was below normal in the domains of physical functioning, physical role and social function, while after vertebral fracture, although physical function, role-physical, bodily pain and social function had improved, all domains were still significantly below reference values [29]. Since there is a lack of data regarding HRQOL from long-term follow-up studies after vertebral or hip fracture, this study was designed to measure HRQOL approximately seven years after vertebral or hip fracture.

The objectives of the present study were to (i) investigate the changes and long-term impact of vertebral or hip fracture and between fracture groups on HRQOL in postmenopausal women prospectively between two and seven years after the inclusion fracture, (ii) compare HRQOL results between fracture and reference groups and (iii) study the relationship between HRQOL and physical performance, spinal deformity index and bone mineral density at seven-year follow-up.

## Methods

### Patient Group

The main inclusion criterion was participation in and completion of the previous two-year follow-up study of women with a newly diagnosed vertebral or hip low-energy fracture, in this paper called the inclusion fracture. The exclusion criteria were refusal to participate and impairment of mental or physical health hindering a subject from providing measurement, correct information and completing the questionnaire.

**Figure 1:**
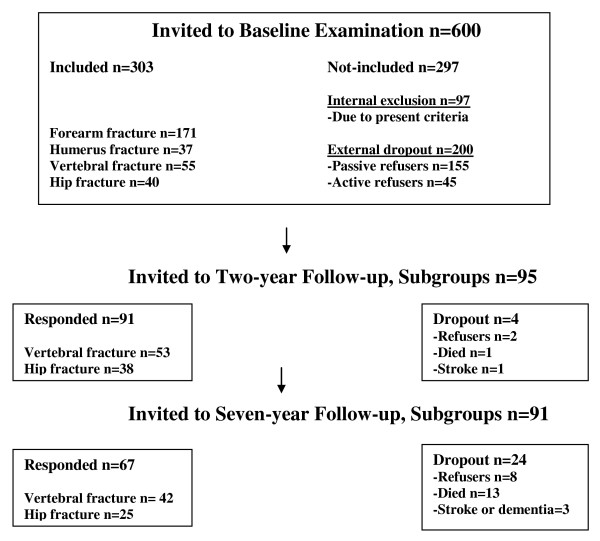
**Flowchart of participant recruitment, inclusion and dropout**.

The participants were invited by phone and post during the period of February through August 2006. The seven-year follow-up was performed a mean of 7.0 years (SD 0.5) after baseline examination. The examinations took place at the University Hospital in Linköping and the osteoporosis unit at Ryhov Hospital in Jönköping, in Sweden. Patients were originally recruited through a written invitation sent to 600 consecutive women with a new low-energy fracture of the distal forearm, proximal humerus, vertebra or hip, as described earlier [29,31]. In the baseline study, 40 women were included after a hip fracture and 55 after a vertebral fracture. The total dropout from baseline to seven-year follow-up was 29% (n = 28). Of these 95 women, seven refused the follow-up visit and three refused the radiological examinations of the spine. Four were excluded due to stroke or dementia, and 14 (three from the hip fracture group and 11 from the vertebral fracture group) had died. Mortality since the two-year follow-up was three in the hip group and ten in the vertebral fracture group. The remaining 67 women were included in the study. A flowchart from baseline to two-year and seven-year follow-up is shown in Figure 1.

A dropout analysis between the missing group (n = 24) and the women (n = 67) participating in the seven-year follow-up, using certain data from the two-year follow-up, showed that the missing group had significantly lower values regarding the SF-36 within the general health and social function domains. They had also lower weight, body mass index and bone mineral density in the hip. Age did not differ.

At two-year follow-up, the patients were prescribed continued osteoporosis medication (usually bisphosphonate, vitamin D and calcium) for the ensuing year, and were referred to their general practitioner for further treatment.

### Reference Group

An age and sex-matched reference group was chosen from a large local population study in Östergötland County, Sweden, called "Östgötens Hälsa 2006", to obtain normative values for HRQOL measured using the Short Form 36 (SF-36) questionnaire. The reference group was handled like a normal background population, recruited from the same general population area during 2006. The SF-36 was mailed to a stratified random sample of 13,440 people aged 18 to 84 years. After two reminders, 7,238 (54%) had responded. For the women aged 65 to 84, 1,144 (68%) had responded. The population study comprised 804 women aged 64 to 82 years, who formed the reference group (mean age 75.7, SD 4.7). From the reference group, women aged 70 and 75 years were compared and showed stable values in all domains, except for vitality, which was significantly lower in the elderly women (59.0 vs. 69.7) [32].

### Design

In this study, a longitudinal design was used to answer the purpose (i) and a cross-sectional for (ii) and (iii). The participants gave their oral informed consent before the visit and written informed consent at the visit. The study was approved by the Regional Ethical Review Board at the Faculty of Health Sciences, University of Linköping 2005, registration no. M173-05, and was performed in accordance with the Declaration of Helsinki.

### Outcomes

The Short Form 36 (SF-36, version 1) of the Medical Outcome Study was used as a main outcome measure of HRQOL [33]. Studied as putative predictors were some specific background data from a self-administered questionnaire, body mass index, physical function evaluated with handgrip strength and one-leg static balance testing, bone mineral density and vertebral fracture assessment.

### Background Data

Before a patient's visit to the osteoporosis unit, a self-administered questionnaire was sent to her containing questions about previous fractures, falls, concomitant diseases, treatments and lifestyle factors (physical activity, calcium intake and smoking) of importance for osteoporosis and fracture risk. A seven-grade scale was used to assess leisure-time physical activity level, modified from the original four-grade scale by Saltin and Grimby [34]. The physical activity levels included household and leisure-time activities. The lowest grade of physical activity was 1, while 7 was denoted as the "high level". A verbal graphic rating scale (GRS) was used to measure present and recalled back pain, for the previous two weeks. The scale used descriptors along a continuum (none-insignificant-mild-moderate-severe-unbearable). Absence of pain was rated as 0 mm, and the worst possible pain as 100 mm [35].

### Short Form 36 (SF-36)

The SF-36 questionnaire was sent to the patient before the visit, and she was asked to answer the questions on her own. If she had not completed the questionnaire before arriving at the unit, she was encouraged to do so before the examination started.

The SF-36 questionnaire comprises 36 items, with two to six response options according to an ordinal scale, assessing eight health concepts or domains: physical function (PF), role limitations due to physical health problems (RP), bodily pain (BP), general health (GH), vitality (VT), social function (SF), role limitations due to emotional problems (RE) and mental health (MH). Each domain allowed a score of 0-100, with a high score indicating better HRQOL. The SF-36 has been evaluated extensively regarding both reliability and validity according to Swedish conditions [36-38].

### Clinical Tests

During the visit, each patient was assessed by the first author (IH). Body height (m) was registered using a stadiometer and body weight (kg) using a calibrated scale. Body mass index (BMI) was calculated using the formula kg/m^2^. Body height and weight were measured in indoor clothes without shoes. Physical function was assessed by measuring handgrip strength and one-leg static balance testing. Handgrip strength (kg) was measured in the dominant hand using the standard JAMAR, an electronic dynamometer. For standardization, the adjustable handle was set at the second position for all women. Participants sat comfortably with their elbow flexed at 90 degrees and their shoulder adducted and neutrally rotated. Each test was performed three times and the mean value was used. Reference values were obtained from Mathiowetz et al. [39] and were adapted to the metric system. The calibration of the instrument was tested periodically during the study. Mathiowetz has recommended the use of the mean of three tests, to achieve the highest test-retest reliability. Static balance was assessed by asking the patients to stand on only their dominant leg with their eyes open. The one-leg-stance tests were performed without shoes with the opposite foot lifted halfway up on the calf of the supported leg and the arms in vertical position. The time was recorded until the supporting foot was moved from its initial position. The static balance tests were timed with a digital stopwatch and were limited to a maximum of 30 s. Static balance tests were performed three times, and the best value on one's dominant leg was used in the final score [40,41].

### Bone Mineral Density

Bone mineral density (BMD) was measured using dual-energy X-ray absorptiometry (DXA, Hologic QDR 4500 A; Hologic Inc., Bedford, MA) of the lumbar spine and hip, non-dominant side. Internal variation was checked regularly with an everyday calibration using a phantom. As a reference for BMD in the hip we used the NHANES III [42] and for BMD in the spine, reference data published by Favus [43].

A patient was classified as osteoporotic if T-score was 2.5 or more standard deviations (SD) below the mean value of young normal (T-score *≤*-2.5) at lumbar spine or hip total, and osteopenic if the lowest of these values was between <-1 and >-2.5 SD [44]. In comparing BMD with the age-matched general population, Z-score was used. Specially trained nurses (DXA operators) performed the measurements.

### Vertebral Fracture Assessment

A lateral digital radiograph of the thoracic and lumbar spine was performed within four months after the visit date, except in one woman who was examined nine months before the visit date. Three women with vertebral fracture as inclusion fracture at the baseline examination refused radiological examination at seven-year follow-up; thus the true incidence of new vertebral fracture is unknown. The number and grade of vertebral deformities were assessed according to the Genant visual semiquantitative criteria [45,46]. Each of the T4 to L4 vertebrae was thus assigned a grade of 0, 1, 2 or 3. Zero indicates no fracture (deformity), 1 a mild (20-25%), 2 a moderate (25-40%) and grade 3 a severe (>40%) height reduction in the anterior, central and/or posterior part, respectively. A Spinal Deformity Index (SDI, range 0-39) was calculated by adding the deformity grades. All the radiological examinations were evaluated by the same experienced skeletal radiologist (SH).

### Statistical Analyses

Group results are reported as mean value (M), standard deviation (SD), confidence interval (CI) and percent. Differences in basic characteristics between vertebral and hip fracture groups were tested using Student's unpaired *t*-test. Pearson's Chi-square was used to analyse categorical data. Missing group bias was analysed by testing the difference between the respondents and non-respondents regarding two-year HRQOL data, using unpaired *t*-tests.

The parametric methods as statistic analytic techniques were chosen in order to adjust for the sampling weights design in the reference group, regarding normative values for SF-36. The reference group was randomly selected from the population registry and weight-adjusted for age to fit with the age distribution for the patient group in this study. For the SF-36, items within each domain were coded, scored and summarized to derive the eight domains. The scores were then translated into a 0-100 scale where 0 indicated the worst possible HRQOL and 100 the best, according to the manual and interpretation guide for SF-36 [47]. SF-36 scores were computed if the respondent answered half or more of the items on the scale; i.e., a person-specific mean score was calculated based on the non-missing items [47]. The analysis included only participants who had completed the seven-year follow-up.

Statistical analyses regarding aim (i) employed Student's paired *t*-test within each fracture group, to measure the change between two-year and seven-year follow-up. To determine the mean value differences between hip and vertebral groups regarding change, we used Student's unpaired *t*-test and ANCOVA while controlling for the effect of covariates, age, new co-morbidity since two-year follow-up and new low-energy fracture since two-year follow-up. We also reassembled the entire group into the groups *new fracture since two-year follow-up *(n = 29), *no new fracture since two-year follow-up *(n = 38), *new co-morbidity since two-year follow-up *(n = 46) and *no co-morbidity since two-year follow-up *(n = 21).

Regarding aim (ii), the SF-36 of the fracture and reference groups were compared using Student's unpaired *t*-test. To determine the differences between hip and vertebral groups we used ANCOVA while controlling for the effect of covariates, age, new co-morbidity since two-year follow-up and new low-energy fracture since two-year follow-up.

With regard to aim (iii), a partial correlation was used in which the relationship is measured, controlling for the effect the covariates have on both variables. Variables in the partial correlation were the eight SF-36 dimensions and static balance on dominant leg with eyes open, handgrip strength on dominant hand, spinal deformity index (SDI), physical activity, bone mineral density in hip total, and fall frequency the past year. The covariates were age, new co-morbidity since two-year follow-up, new low-energy fracture since two-year follow-up (dichotomous variables, yes = 1 or no = 2) and fracture group (vertebral = 1 hip = 2). Differences were defined as significant if the level of *p*-value was < 0.05 (2-sided) [48]. All statistical analyses were performed using SPSS^® ^for Windows version 15.0 (Statistical Package for the Social Sciences, SPSS Inc., Chicago, IL).

## Results

### Patient Characteristics

Of the 67 patients in the total study group, 42 had suffered a vertebral fracture and 25 a hip fracture shortly before the baseline study (inclusion fracture). The mean age (SD) of the entire study group was 75.5 (4.6), range 64-82 years at seven-year follow-up. A total of 51% were married or cohabiting.

At seven-year follow-up, 29/67 women had sustained one or more new clinical low-energy fractures, in total 49 fractures since two-year follow-up. More patients in the vertebral group (22/42), than in the hip fracture group (7/25), had sustained a new clinical fracture. In the hip fracture group at seven-year follow-up, nine women were identified as having one or more vertebral fractures. Six of these women had no previous thoracolumbar radiographs, and the true baseline prevalence and the seven-year incidence of vertebral fracture in this group are unclear. In one woman the vertebral fractures were known before the inclusion, and two women had new vertebral fractures compared with the previous radiographs.

Back pain during the past 14 days was reported to be disturbing (GRS >30 mm) by 36/42 in the vertebral fracture group and by 15/25 in the hip fracture group, a significant difference (*p *= 0.02). In the vertebral fracture group, 48% took painkillers (analgesics) regularly, 24% sometimes and 28% never. In the hip fracture group, 32% took painkillers regularly, 20% sometimes and 48% never, a non-significant difference (*p *= 0.263). The most frequently used painkillers were paracetamol (93%), opioids (44%) and NSAID (41%) alone or in combination, regularly or as required.

Bisphosphonate treatment was currently being used by 34% women, 15/42 and 8/25 respectively, *p *= 0.76. Most patients took a supplement of calcium in combination with vitamin D, 86% in the vertebral group and 70% in the hip group. Seven were active smokers (10%). Overall, 69% women reported one or more new co-morbid conditions of greater importance since two-year follow-up. The incidence of new co-morbidity did not differ between the fracture groups. The total number of reported new co-morbid conditions was 71 (42 in the vertebral group and 29 in the hip group), the most frequent being cardiac disease, 25 (18 and 7, respectively), rheumatic or musculoskeletal, 15 (9 and 6, respectively) and bronchi-pulmonary disorders, 7 (3 and 4, respectively). Further basic characteristics of the two fracture groups are presented in Table 1.

**Table 1:** Characteristics of participants, by fracture group, at seven-year follow-up

	**Vertebral group** **n = 42**	**Hip group** **n = 25**	***p*-value**
Age, yr, mean (SD)	75.8 (4.7)	75.0 (4.7)	0.50
Weight (kg) (SD)	69.5 (16.1)^1^	70.0 (10.8)	0.91
Height (cm) (SD)	158.4 (7.0)^1^	162.1 (5.5)^1^	**0.03**
Body mass index, mean (SD)	27.5 (5.0)^1^	26.6 (4.0)^1^	0.48
Static balance, one-leg second (SD)	8.9 (10.5)^2^	7.0 (8.3)	0.43
Handgrip, kilo, mean (SD)	16.7 (6.6)^2^	19.8 (5.0)	**0.04**
Back pain (GRS), mean (SD)	52 (22)	38 (27)	0.36
SDI (SD)	7.8 (6.1)^2^	2.3 (4.2)^1^	**<0.01**
BMD Lumb, Z-score SD	0.49^4^	0.73^2^	0.52
BMD Hip total, Z-score SD	-0.18^3^	-0.43^2^	0.38
BMD Femoral neck, Z-score SD	-0.05^3^	-0.35^2^	0.22
Participants with new co-morbidity since 2-year follow-up, %	67	72	0.65
Fall last year, %	38	32	0.62
Participants with new clinical fracture since 2-year follow-up, %	52	28	**0.05**
Total number new clinical fracture since 2-year follow-up (n)	36	13	**-**
Distal forearm (n)	3	2	**-**
Proximal humerus (n)	2	1	**-**
Hip (n)	6	1	**-**
Vertebrae (n)	16	2	**-**
Other (n)	9	7	**-**

Significance level p < 0.05 is given in bold.

Missing individuals ^1 ^= 1, ^2 ^= 2, ^3 ^= 3, ^4 ^= 7.

### SF-36 Longitudinal Change - between two and seven-years

The vertebral fracture group had no statistically significant changes in any SF-36 domains except bodily pain, which had decreased significantly at seven-year follow-up, indicating increased pain. The hip fracture group had no significant changes in any domains (Table 2). However, between the fracture groups there were no significant mean value differences in the change between two and seven years, or after controlling for covariates, age, new co-morbidity and new fracture.

**Table 2:** HRQOL (SF-36) mean values in different fracture groups at two-year and seven-year follow-up

**SF-36**	**Vertebral** **Two-year**	**group** **Seven-year**	**n**	*p*-value*	**Hip** **Two-year**	**group** **Seven-year**	**n**	*p*- value*
**PF**	52.2 (21.8)^a^	50.2 (26.2)	41	*p *= 0.567	57.1 (22.1)	58.2 (21.1)	25	*p *= 0.805

**RP**	38.5 (41.4)	26.9 (38.5)	40	*p *= 0.110	58.0 (44.3)	41.0 (39.4)	25	*p *= 0.124

**BP**	49.7 (23.8)	41.6 (23.7)	41	***p *= 0.022**	64.5 (24.0)	60.6 (26.7)	25	*p *= 0.477

**GH**	56.1 (23.5)	56.1 (24.3)	40	*p *= 0.983	69.9 (20.5)	65.4 (17.6)	25	*p *= 0.230

**VT**	47.5 (23.6)	47.4 (25.1)	41	*p *= 0.991	66.2 (23.1)	63.2 (18.1)	25	*p *= 0.424

**SF**	73.8 (25.7)	66.2 (28.1)	41	*p *= 0.085	84.5 (18.8)	83.0 (18.4)	25	*p *= 0.718

**RE**	65.0 (43.3)	54.2 (45.1)	40	*p *= 0.230	76.0 (39.1)	80.0 (33.3)	25	*p *= 0.559

**MH**	71.6 (22.8)	70.9 (22.6)	40	*p *= 0.415	79.4 (16.4)	82.7 (12.7)	25	*p *= 0.287

PF: Physical Functioning, RP: Role-Physical, BP: Bodily Pain, GH: General Health,

VT: Vitality, SF: Social Functioning, RE: Role-Emotional, MH: Mental Health.

^a ^Values in parentheses correspond to standard deviations.

*Significant tests: paired *t*-test

*p*-value: longitudinal change since two-year follow-up in different fracture groups

Significance level p < 0.05 is given in bold.

The group with new fracture (n = 29), of whom 22 belonged to the vertebral group, had significantly lower values at seven-year follow up regarding role-physical, bodily pain, general health and social function (all p < 0.01). The group with no new fracture (n = 38), of whom 20 belonged to the vertebral group, had no significant changes. The group with new co-morbidity (n = 46) had no significant changes, and neither the group without new co-morbidity (n = 21).

### SF-36 at Seven-year Follow-up

The vertebral fracture group had significantly lower scores than the reference group in all domains, except for general health and mental health.

Women with hip fracture did not differ from the reference group regarding any SF-36 domain, but better values were found for their mental health (Table 3).

**Table 3:** HRQOL (SF-36) mean values in reference and different fracture groups at seven-year follow-up

**SF-36**	**Reference group** **n = 804**	**Vertebral group** **n = 42**	**Hip group** **n = 25**	*p*-value*
**PF**	62.7 (26.7)^a^	50.4 (25.9)	58.2 (21.1)	***p***1 = **0.004 ***p*2 = 0.404

**RP**	55.9 (44.9)	27.4 (37.8)	41.0 (39.4)	*p*1<**0.001 ***p*2 = 0.075

**BP**	59.0 (27.5)	41.6 (23.5)	60.6 (26.7)	*p*1<**0.001 ***p*2 = 0.777

**GH**	58.2 (22.8)	55.8 (24.4)	65.4 (17.6)	*p*1 = 0.515 *p*2 = 0.055

**VT**	58.0 (25.4)	48.0 (25.1)	63.2 (18.1)	*p*1 = **0.013 ***p*2 = 0.173

**SF**	77.3 (26.3)	67.0 (28.3)	83.0 (18.4)	*p*1 = **0.013*** p*2 = 0.145

**RE**	71.3 (40.6)	54.0 (45.4)	80.0 (33.3)	*p*1 = **0.020 ***p*2 = 0.212

**MH**	74.7 (21.0)	71.2 (22.3)	82.7 (12.7)	*p*1 = 0.296 ***p***2 = **0.005**

PF: Physical Functioning, RP: Role-Physical, BP: Bodily Pain, GH: General Health, VT: Vitality,

SF: Social Functioning, RE: Role-Emotional, MH: Mental Health.

^a ^Values in parentheses correspond to standard deviations.

*Significant tests: unpaired *t*-test

*p*1 comparison between vertebral and reference groups

*p*2 comparison between hip and reference groups

Significance level p < 0.05 is given in bold.

The vertebral fracture group had significantly lower values for bodily pain, vitality, role-emotional function and mental health compared to the hip fracture group after controlling for covariates, age, new co-morbidity and new fracture (Table 4).

**Table 4:** HRQOL (SF-36) adjusted mean values in vertebral and hip fracture groups at seven-year follow-up

**SF-36**	**Vertebral group** **n = 42**	**Hip group** **n = 25**	*p*-value*
**PF**	51.6 (44.5-58.8)^a^	56.0 (46.7-65.4)	*p *= 0.464

**RP**	30.5 (20.0-41.1)	35.7 (21.8-49.5)	*p *= 0.563

**BP**	42.9 (36.1-49.6)	58.5 (49.7-67.3)	***p *= 0.007**

**GH**	56.2 (49.3-63.1)	64.9 (55.9-73.9)	*p *= 0.136

**VT**	48.5 (42.3-54.7)	62.3 (54.2-70.4)	***p *= 0.010**

**SF**	68.5 (61.1-75.9)	80.4 (70.7-90.0)	*p *= 0.060

**RE**	54.7 (42.0-67.3)	78.9 (62.3-95.4)	***p *< 0.026**

**MH**	71.2 (65.4-77.1)	82.7 (75.0-90.4)	***p *< 0.023**

PF: Physical Functioning, RP: Role-Physical, BP: Bodily Pain, GH: General Health,

VT: Vitality, SF: Social Functioning, RE: Role-Emotional, MH: Mental Health.

^a ^Values in parentheses correspond to 95% confidence intervals.

*Significant tests: ANCOVA, means controlling for the effect of covariates, age, new co-morbidity, and new fracture since two-year follow-up.

Significance level p < 0.05 is given in bold.

Regarding differences between vertebral and hip groups, the covariate age was significantly related to physical functioning, role-physical, bodily pain and vitality. The covariate new co-morbidity was significantly related to role-physical, bodily pain, vitality and role-emotional. The covariate new fracture was significantly related to role-physical, bodily pain and social functioning.

### Clinical Tests

Handgrip strength was significantly better in the hip fracture group, 19.8 (SD 5.0), compared with that of patients with vertebral fracture, 16.7 (SD 6.6). Static balance, standing on one's dominant leg with one's eyes open, did not differ between the fracture groups. Body height was significantly higher in the hip fracture group. Height loss did not differ significantly between the groups (*p *= 0.63), with a mean loss since baseline visit of 21 mm (SD 19) in the vertebral fracture group and 19 mm (SD 16) in the hip fracture group. Further basic characteristics of the two fracture groups are shown in Table 1.

### Bone Mineral Density

Bone mineral density (BMD) did not differ significantly between the vertebral and hip fracture groups. According to the WHO criteria [44], among women with vertebral fracture, 41% had osteoporosis (*T*-score *≤*-2.5), 54% had osteopenia/low BMD (*T*-score <-1 and >-2.5) and 5% normal value (*T*-score *≥*-1.0) in the hip and/or spine. In the hip fracture group, 50% had osteoporosis, 42% had osteopenia/low BMD and 8% normal value. Additional BMD Z-score data are presented in Table 1.

### Vertebral Fracture Assessment

According to the lateral radiographs of the spine in the present seven-year follow-up, 51 women had one or more vertebral fractures (nine of whom originally had a hip fracture as inclusion fracture). Sixteen women in the hip fracture group had no vertebral fracture. In the group with vertebral fracture(s), SDI was 7.8 (6.1 SD) and range 1-25, and in the hip fracture group SDI was 2.3 (4.2 SD) and range 0-15. Seventeen patients had one vertebral fracture and eight patients had two fractures, and as many as 48% of the women had three to nine vertebral fractures. Two patients had nine vertebral fractures, SDI 23 and 25, respectively.

### Partial Correlations

In the total fracture group (n = 67), physical activity correlated positively with all domains except the role-emotional one, static balance showed a significantly positive correlation to most of the SF-36 domains, except social function and role-emotional function. Also, handgrip strength showed a significantly positive correlation to role-physical, vitality and mental health. Fall frequency showed negative correlation with bodily pain and vitality. BMD in the hip and SDI was not significantly correlated with SF-36. These data show the relationship between the two variables, controlling for the effect of covariates (Table 5).

**Table 5:** Partial correlation of: SF-36 domains vs. static balance, handgrip strength, SDI, BMD hip total, fall frequency and physical activity, at seven-year follow-up

	**Static balance** **n = 60**	**Handgrip strength** **n = 60**	**SDI** **n = 60**	**BMD hip tot** **n = 60**	**Fall frequency** **n = 60**	**Physical activity** **n = 60**
**SF-36:**						
**PF**	0.48***	0.17	-0.02	0.02	-0.16	0.72***
**RP**	0.50***	0.28*	-0.05	0.22	-0.09	0.45***
**BP**	0.27*	0.22	-0.05	-0.10	-0.29*	0.37**
**GH**	0.36**	0.25	-0.03	0.00	-0.24	0.35**
**VT**	0.42**	0.39**	-0.11	0.06	-0.35**	0.37**
**SF**	0.19	0.14	-0.10	-0.01	-0.19	0.30*
**RE**	0.21	0.17	-0.21	0.13	-0.26	0.16
**MH**	0.29*	0.29*	-0.00	0.13	-0.22	0.31*

PF: Physical Functioning, RP: Role-Physical, BP: Bodily Pain, GH: General Health,

VT: Vitality, SF: Social Functioning, RE: Role-Emotional, MH: Mental Health.

Partial correlations, controlling for the effect of age, fracture group, new co-morbidity and new fracture since two-year follow-up.

Significance level p < 0.05 is given in bold. *p < 0.05 **p < 0.01 ***p < 0.001

## Discussion

The present study, which to our knowledge is the longest published prospective follow-up study regarding HRQOL after vertebral or hip low-energy fracture in routine health care, supports and provides more details to the hypothesis that vertebral fractures have a severe long-term impact on HRQOL, assessed using the SF-36.

The vertebral group scored lower than did the reference group in most domains at seven-year follow-up. Also, bodily pain had deteriorated between two and seven years and might be explained by new fracture. The total group with new fracture since two-year follow up also had lower values at seven-year follow-up, which can be interpreted as the vertebral and hip fracture groups with subsequent fractures (all fractures, not only new vertebral ones) having poorer self-rated health, measured using the SF-36 at seven-year follow-up. A recent study with five years of prospective data about the long-term impact of incident fractures on HRQOL supports these findings [30]

The hip fracture group had stable values in all domains between two and seven years. Regarding the hip fracture group, it should be noted that the mean age of the patients was 68 yr at the time of the fracture, and most of them were able to return to active life. Hip fracture at this relatively early age is thus mainly a transient problem for the patient in contrast to the case of vertebral fracture. The hip fracture group, despite the incident or prevalent vertebral fracture(s) (mainly subclinical fractures) in nine women, did not differ from the reference group regarding HRQOL after seven years, and even had better values for mental health.

In this study the vertebral fracture group had lower scores than the hip fracture group in bodily pain, vitality, role-emotional function and mental health at seven-year follow-up after controlling for age, new fracture and new co-morbidity.

These findings support the suggestions from previous cross-sectional studies that pain and disability after vertebral fracture do not fade away [4,24,25] unless effective treatment is given [20,21].

The patients were prescribed, and most took, treatment (usually bisphosphonates, calcium and vitamin D) for the first three years, but at seven-year follow-up only 34% were still on active anti-osteoporosis treatment, and their true compliance is not known. Is this due to a lack of continued prescription or to non-compliance by the patients?

The effect of more intensive treatment strategies should be evaluated further, for both pharmacological and non-pharmacological treatments. Increased bone mineral density and reduced fracture incidence are a good start, but patients' well-being also has to be taken into account. According to current recommendations, all patients still fulfilled the criteria for active anti-osteoporosis treatment [49].

The partial correlations show relationships between HRQOL and physical activity, static balance, and handgrip strength, and in some domains fall frequency, despite controlling for covariates such as age, fracture group, new fracture and new co-morbidity. Static balance, expressed as the ability to stand on one leg with one's eyes open, is often used as a clinical test of balance, and is considered to be sensitive to age-related changes in balance [41] and an important predictor of injurious falls in older people [50]. Bohannon et al. [41] found that participants aged 70-79 years (n = 31, men and women) could maintain this position for a mean of 14 s. Healthy Swedish women aged 70 years held this position for a mean of 18 s [40]. The women in this study maintained their balance while standing on their dominant leg with their eyes open for an average of 8 s; this low value suggests an impairment of static balance. Handgrip strength was significantly lower in the vertebral fracture group than in the women with hip fracture. The mean values in the hip fracture group did not differ from the reference values in American women of the same mean age [39]. A recent population study from Sweden assessed handgrip strength in 75 and 80-year-old women. Handgrip strength was measured using the JAMAR, with the test being repeated three times and the highest value being recorded. In women at risk for malnutrition, handgrip strength was 19.7 kg and in women at no risk 23.5. Compared to this study, the vertebral group had lower handgrip strength (16.7) with both groups for risk and no risk for malnutrition. The handgrip strength in the hip fracture group (19.8) is at the level of women at risk for malnutrition [51]. Patients with hip fracture are found to be thinner with lower lean body mass than age-matched controls [52].

Dixon et al. (2005) found an association between low handgrip strength and low bone mineral density and an increased risk of incident vertebral fracture in the European Prospective Osteoporosis study (EPOS) [53].

The results support the value of the SDI [45] as a measure to be used in clinical routine. This approach is more objective and reproducible than a visual qualitative assessment of vertebral fracture [46]. Accurate radiographic diagnosis is important, as the underdiagnosis of vertebral fractures may lead to decreased rates of diagnosis and treatment of osteoporosis in women [8].

In the present group, as many as 29 of 67 (43%) had one or more new fractures during the five-year period (since two-year follow-up). This high fracture incidence may be ascribed to the degree of osteoporosis as well as to the less ambitious treatment.

A prospective three-year study showed that for each new vertebral deformity, HRQOL deteriorated further [28]. Similar results have been reported by others [7,10,26].

The question of whether the poor HRQOL and the poor survival in patients with vertebral fracture are actually due to the fractures, increased biological age or concomitant diseases is important and may be pivotal to attitudes regarding osteoporosis treatment [9,11,12]. Recent results from a three-year controlled study with an annual intravenous injection of zoledronate showed not only reduced fracture incidence but also an increased survival [54]. These studies support the hypothesis that osteoporosis and fracture may be causative factors of chronic back pain and excess mortality. Mortality is reported to increase after vertebral as well as hip fracture [9-12]. The reduced physical function, higher incidence of new fractures and higher mortality in our vertebral fracture group likewise suggest that vertebral fracture predicts a poorer prognosis than a hip fracture does in this age group. The causes of excess mortality after vertebral fractures are still obscure, although cancer and pulmonary death have been suggested [11]. The findings of reduced handgrip strength and static balance in the vertebral fracture group may support a relationship with general frailty. But which is the chicken and which is the egg?

Approximate expected mortality during the five-year period (since the two-year follow-up) for the two fracture groups was assessed on age-specific death risks in Sweden 2006 [55]. The age-adjusted number of expected deaths in the group with vertebral fracture (n = 53) and hip fracture (n = 38) was five (CI 2-9) and four (CI 1-7), respectively. Five-year mortality in the vertebral fracture group (10/53) was high, but the study was not designed to identify causes of excessive death rates.

The results of this study thus support previous reports that vertebral fractures are associated with increased pain, impaired physical function [4,7], decreased HRQOL [26,28,30] and higher morbidity and mortality [9,10,12].

Pharmacological as well as non-pharmacological treatments and methods have to be considered. Malmros et al. (1998) found positive effects from a ten-week ambulatory exercise programme with physiotherapy on chronic pain, balance, physical function and quality of life [56]. There is a lack of studies and evidence of non-pharmacological treatments effects assessed with self-reported quality of life questionnaires after osteoporosis fracture. The development of strategies to improve HRQOL after vertebral fracture remains an important goal for future research.

Some methodological issues need to be considered. Multiple testing increases the risk of obtaining a significant difference purely by chance; therefore, the results should be interpreted with caution. However, if a simple sequentially rejective multiple test procedure (Holm's method) had been used [57], most of the results in the study would still be significant, because most had a p < 0.01.

For the most part, our results show clinically significant differences. A half standard deviation is a conservative estimate of clinical significance, but the minimally important difference may be below 1/2 SD in specific cases [58].

Many patients adapt over time, and their perceptions of HRQOL may change. Learning to cope with problems is a well-recognized characteristic of the chronically ill. Also, patients may meet others whose condition is worse or better than their own, which can lead to a revaluation of their own internal standards and values.

Response shift is a psychological phenomenon that results from coping caused by the affecting internal standards or values [59]. A response shift cannot be excluded in this study; the HRQOL response patterns could have been affected by choice of comparator group. A study conducted by Fayers et al. found that the majority of questionnaire participants reported using different frames of reference for comparison when completing an annual HRQOL questionnaire [60]. Only one-third of the participants reported using the same comparison reference frame at each yearly interval.

In this study, we chose to use a generic HRQOL questionnaire instrument, to be able to make comparisons with a reference group. It is possible that the outcome would have been different if we had used an osteoporosis-specific questionnaire on HRQOL. However, at the start of the baseline study there was no disease-specific questionnaire available that had been translated into Swedish and validated.

A limitation of the study is the small number of women, particularly in the hip fracture group. A baseline radiograph of the lumbar and thoracic spine was performed for only one woman with a hip fracture as inclusion fracture and, therefore, the true prevalence of vertebral fractures in this group at baseline is unclear.

The reference group was handled like a normal background population, and we lacked data concerning co-morbidity and fracture status, which would have been advantageous to have in order to adjust for possible covariates. The missing group of 26% since two-year follow-up could have affected the result. A dropout analysis between the missing group and the women participating in the seven-year follow-up, using data from the two-year follow-up, showed that the missing group had significantly lower values regarding the SF-36 as well as lower weight, body mass index and bone mineral density in the hip. This can be interpreted as the missing group having poorer health, and the result at seven-year follow-up possibly leading to an overestimation of SF-36 scores for the vertebral and hip group.

The low doctor compliance with treatment may be regarded as a limitation, but indeed allows the study expose the symptom development in a poor-treatment or non-treatment situation. We studied only Caucasian women, and the results may not apply to other ethnic groups or men.

Strengths of the study are its prospective design and the fact that all participants were investigated using well defined methods. The advantages of using the SF-36 were the possibility to assess and compare HRQOL in individuals suffering from different co-morbidity and supplying reference data for the general population. Another strength is the large reference group for the SF-36 recruited from the same general population during 2006.

## Conclusion

This study demonstrates that women who had had vertebral fracture as inclusion fracture had remaining pronounced reduction of HRQOL at seven-year follow-up. A decreased HRQOL since the two-year follow-up might be explained by new fracture.

In the age span of 64-82 years (mean age 75.5), the prevalence of vertebral fracture suggests more negative impact on HRQOL, more severe osteoporosis and a poorer prognosis than a hip fracture does. The differences in HRQOL between vertebral and hip fracture at seven-year follow-up cannot be explained by age, new disease or new fracture. Women with hip fracture did not differ from the reference group regarding HRQOL, despite vertebral fractures in nine women.

The long-term reduction of HRQOL and its relationship to physical activity, static balance, and handgrip strength raise questions that warrant more investigation. Furthermore, HRQOL studies with more effective treatment including non-pharmacological intervention are needed, and the development of strategies to prevent loss of function and improve HRQOL after vertebral fracture remains an important goal for future research.

## Competing interests

The authors declare that they have no competing interests.

## Authors' contributions

IH participated in the design of the study, conducted patient recruitment, collected the data, performed the statistical analyses, and clinical evaluation, and drafted and revised the manuscript. M B-L participated in the design of the study, analysis and interpretation of data, statistical and clinical evaluation, and the progress and revision of the manuscript. SH participated in the design of the study regarding vertebral fracture assessment and evaluated all the radiographs. GT participated in the design of the study, analysis and interpretation of data, clinical evaluation, and the progress and revision of the manuscript. A-C E participated in the design of the study, analysis and interpretation of data, statistical and clinical evaluation, progress and revision of the manuscript, and served as project supervisor. All authors read and approved the final manuscript.

## Pre-publication history

The pre-publication history for this paper can be accessed here:


